# Evaluation of awareness and performance towards COVID-related disinfectant use: a comparative study between Jordan and United Arab Emirates

**DOI:** 10.1186/s12879-024-09447-1

**Published:** 2024-06-05

**Authors:** Wissam Ghach, Aseel A. Takshe, Mohammad Rababa, Sami Al-Rawashdeh, Nisreen Alwan

**Affiliations:** 1 Department of Public Health, Canadian University Dubai, Dubai, United Arab Emirates; 2 Faculty of Nursing, Jordan University of Science and Technology, Irbid, Jordan; 3 Department of Community and Mental Health Nursing, Faculty of Nursing, The Hashemite University, P.O. Box 330127, Zarqa, 13133 Jordan; 4 College of Health Sciences, Abu Dhabi University, Abu Dhabi, United Arab Emirates

**Keywords:** COVID-19, Awareness, Performance, Disinfectant use, UAE, Jordan

## Abstract

**Background:**

The World Health Organization recommended the use of chemical-based disinfectants as an effective prevention of the COVID-19 pandemic. However, calls for poisoning were reported in several medical centers. The widespread use of chemical-based disinfectants as a preventive measure during the COVID-19 pandemic has underscored potential gaps in community awareness and performance, posing health risks. This study evaluates and compares levels of awareness and performance regarding the safe use of disinfectants in Jordan and UAE.

**Methods:**

The study was conducted between October 2022 and June 2023 via an online questionnaire. Data of respondents from Jordan (*n* = 828) and UAE (*n* = 619) were analyzed using SPSS. ANOVA, Mann-Whitney, and Kruskal-Wallis tests evaluated significant differences in awareness and performance levels across different demographic groups in Jordan/UAE and between them. Spearman’s correlation test examined the correlation between awareness and performance among respondents. Multinomial logistic regression analysis explored associations between various variables and awareness/performance levels within each population.

**Results:**

Findings reveal weak awareness (72.4% and 9.03% in UAE and Jordan, respectively) and moderate performance level (98.8% in UAE and Jordan), with a weak correlation (UAE, rho = 0.093; Jordan, rho = 0.164) observed between the two countries (*P* < 0.05). Multinomial logistic regression analysis indicates gender-related associations with awareness levels and education-related associations with performance levels.

**Conclusions:**

The study emphasizes the urgent need for awareness campaigns and workshops to promote safer disinfectant practices to develop effective interventions aligning with sustainable development goals.

**Supplementary Information:**

The online version contains supplementary material available at 10.1186/s12879-024-09447-1.

## Introduction

In December 2019, an outbreak of new pneumonia named “Severe Acute Respiratory Syndrome Coronavirus-2 (SARS-CoV-2)” or ”Coronavirus Disease 2019 (COVID-19)” was reported in Wuhan, China [1]. According to the fast spread of SARS-CoV-2 in several countries, the World Health Organization (WHO) classified it as a global pandemic on March 11th, 2020 [2]. The clinical investigations of COVID-19 show that fever, diarrhea, nausea or vomiting, fatigue, headache, body aches, loss of taste or smell, congestion or runny nose, sore throat, and cough are the common symptoms of COVID-19 infection [3]. Moreover, the main modes of COVID-19 transmission are human-to-human interactions and human-to-object interactions, especially when solid surfaces are contaminated by respiratory droplets and aerosols of COVID-19 patients [4, 5]. The latter can be reduced via public commitment to the WHO preventive measures in public areas such as mask use, physical distancing, skin hygiene and disinfection especially after touching any dubious surfaces [5].

For an effective prevention of the COVID-19 pandemic, WHO recommended the use of chemical-based disinfectants (i.e., ethanol, isopropanol, chlorine-containing disinfectants, benzalkonium chloride, quaternary ammonium compounds, and hypochlorite) for skin and/or surface disinfection according to the Centers for Disease Control and Prevention (CDC) guidelines [6]. According to the limited public awareness of chemical safety, calls for poisoning were reported in several medical centers during the spread of the pandemic [7–9]. Responding to these worldwide calls, an investigation found that the US public community had poor awareness regarding the safe use of cleaning-disinfection products and accordingly high-risk practices at the household settings [10]. Additionally, the household settings in Abu Dhabi [11] and Lebanon [12] recorded poor awareness along with the occurrence of at least one irritation-to-poisoning symptom during the use of disinfectants and cleaning products. Similar studies showed weak awareness regarding the safe use of personal disinfectants among the public community in Qom, Iran [13] and the university communities in Lebanon [14]. According to these studies, public awareness, practices, and sources of information were interrelated variables that may affect the safe handling of chemical disinfectants among the public communities [11, 13, 14]. To the best of our knowledge, limited/no published studies had compared the awareness and performance regarding the use of chemical disinfectants among the public community in Middle Eastern Arab countries specifically in Jordan and UAE.

During the spread of the pandemic, the Middle Eastern Arab communities were expected to record a high incidence of COVID-19 infections as a result of religious activities (e.g. pilgrimages and regular prays in mosques and churches), high cultural diversity in the UAE [15], and lack of humanitarian quality in Syrian refugee camps in Jordan [16]. According to the WHO statistics, UAE had over 1 million confirmed cases of COVID-19 with 2,349 deaths while Jordan had over 1,700,000 confirmed cases of COVID-19 with 14,122 deaths [17, 18]. Since the onset of the pandemic, the local governmental authorities (Jordanian Ministry of Health and UAE Ministry of Health and Prevention) have enforced COVID-19 preventive measures based on the WHO guidelines to limit the spread of SARS-CoV-2 and consequently reduce the impact of COVID-19 on public health in Jordan and UAE. Moreover, public communities require a full awareness to use personal disinfectants in safe and effective protocols of SARS-CoV-2 prevention. Despite of such alarming expectations and encouragement, limited studies have investigated the community levels of awareness and practices regarding the safe use of chemical-based disinfectants during the COVID-19 period in Jordan and UAE [11, 19]. In the Emirate of Abu Dhabi, Alwan et al. (2023) evaluated public awareness and performance regarding the use of household cleaning products and disinfectants. The study focused on household settings and did not include other emirates of the UAE [11]. In the North, South, and Central regions of Jordan, Bardaweel et al. (2023) assessed the general knowledge and practices regarding disinfectants and sanitizers. The aforementioned Jordanian study focused on the safety instructions and preventive measures during the use of disinfectants and sanitizers rather than the ingredients, preparation, and handling protocols of chemical-based disinfectants [19]. Public communities require a full awareness to use personal disinfectants in safe and effective protocols of SARS-CoV-2 prevention. Additional studies focused on the awareness, attitude, and practices regarding COVID-19, diagnostic symptoms, side effects, sources of information, and general preventive measures (e.g. mask use, physical distancing, skin hygiene, and quarantine) among communities in Jordan and UAE [20, 21]. Applying such a comparative study is highly recommended to assess the safe use of chemical-based personal disinfectants for the current and future pandemics in the Middle Eastern Arab countries.

To the best of our knowledge, the levels of awareness and performance toward personal disinfectant use in Jordan and UAE have not been investigated based on the international CDC and WHO safety guidelines. For the first time, this paper aims to evaluate the awareness and performance levels regarding the safe use of personal disinfectants among the public communities in Jordan and UAE. The expected outcome of this study would reduce the awareness gap and emphasize the safe use of chemical-based disinfectants through community-based interventions and awareness campaigns in these countries. Local governmental authorities, non-governmental organizations, healthcare professionals, and public health researchers would utilize the study outcomes as up-to-date evidence to design new research and awareness initiatives tackling the safe use of hygienic chemicals for current and future pandemics in developing countries.

## Methods

A cross-sectional study was conducted between October 2022 and June 2023 to assess the levels of awareness and performance regarding the safe use of personal disinfectants in Middle Eastern Arab countries (Jordan and UAE). Using a convenience sampling technique, an online google survey was distributed throughout social media platforms (i.e., Facebook, Instagram, Twitter, and LinkedIn), and research offices/centers of the academic institutions in Jordan and UAE. In order to reduce sampling bias, the questionnaire was circulated, as stated above, to a broader segment of our target population using a multiple channels for recruitment (e.g., social media, email lists, Facebook, etc.) to capture a more diverse range of respondents.

### Study population

The study population was divided into two based on the geographical location of the respondents. Population A included 828 respondents, aged more than or equal to 18 years old and participated electronically, from the twelve Governorates of Jordan. Population B included 619 respondents, aged more than or equal to 18 years old and participated electronically, from the seven UAE emirates. Youngsters aged less than 18 years old were excluded from this study. Each population was then stratified into groups based on gender, age, educational level, and place of residence per Governorate or Emirate.

### Study tool

In this study, the validated questionnaire was adapted from the Iranian study of disinfectants (supporting information) [13]. The study tool is already pre-tested by a panel of experts from the School of Health, Qom University of Medical Sciences and validated (through content validity ratio (CVR) and content validity index (CVI)).

The questionnaire was divided into four sections: (1) five items representing profile demographics (age, gender, educational level, governorate/emirate, and previous experience with SARS-CoV-2 infection); (2) a multiple-choice item representing the resources of COVID information (Personal experience, internet webpages, television (TV), social media platforms, healthcare professionals, governmental health authorities, and the webpages of CDC and WHO); (3) 10 True/False items to measure the community awareness regarding the safe handling of disinfectants; and (4) 18 Yes/No and Likert-type items (Never, rarely, sometimes, most of the time, always) to measure the community performance regarding the use of disinfectants among the two studied populations. The grading system (0 and 1 for incorrect and correct responses, respectively) was applied to stratify the community awareness into intervals of weak [0, 4], moderate [5, 7], and good [8–10] based on the mean score of each population. Similarly, the grading system (0 for no and never, 1 for yes and rarely, 2 for sometimes, 3 for most of the time, and 4 for always) was applied to stratify the community performance into intervals of weak [0, 20], moderate [21, 40], and good [41–57].

### Data analyses

Data analysis was conducted using IBM SPSS version 28 (SPSS, International Business Machine Corp. IBM, Chicago, IL, USA). To portray the sociodemographic characteristics of the respondents, percentage frequencies were calculated and presented in tables for each of the studied population. Median values, along with the interquartile range (IQR), were computed and tabulated to summarize the levels of awareness and performance within the two studied populations. Tests for normality and homogeneity of variances were performed using Kolmogorov-Smirnov/Shapiro-Wilk and Levene’s tests, respectively, for all variables. Violations of these assumptions (*P* < 0.05) led to the use of Mann-Whitney tests or Kruskal-Wallis tests, instead of t-tests or One-way ANOVA, as appropriate. Specifically, Mann-Whitney tests were employed to identify significant differences in performance and awareness levels concerning gender within each of the two populations. Kruskal-Wallis tests were utilized to assess significant differences in awareness and performance levels for other variables.

To explore the correlations between respondents’ awareness and performance regarding the use of disinfectants, Spearman correlation tests were employed within each of the studied population. Furthermore, a multinomial logistic regression analysis was performed to investigate the association between the variables mentioned above (gender, age, governorate/emirate, and educational level) and the levels of awareness and performance within each studied population. Additionally, a univariate ANOVA was conducted to examine significant differences of awareness and performance levels in the studied variables between population A and population B (Gender, age group, and educational level only as the emirate and governorate cannot be compared). All data analyses were conducted at a significance level of 0.05, with *P* < 0.05 indicating statistical significance and confidence intervals set at 95%.

### Ethical considerations

The study protocol was approved by the Institutional Review Board (IRB) at Abu Dhabi University (ADU) (CAS– 22-08-00023), and the Hashemite University (2/9/2021/2022). In the first page of the google survey, an informed consent was used to identify the purpose, risks, benefits, and confidentiality of the study. At the end of the informed consent, a statement was added to stress that participation is voluntary, and that the questionnaire submission indicates the individual’s approval to participate in the study.

## Results

Table 1 shows the frequencies and percentages of the study population demographics in Jordan and UAE during the spread of COVID-19. The mean ages of the study population were (26.41 ± 8.95) years in population A, and (28.15 ± 10.93) years in population B.

**Table 1:** Frequencies and percentages of the study population demographics in Jordan and UAE during the spread of COVID-19

Variables (Population A: Jordan)	Frequency	Percentage	Variables (Population B: UAE)	Frequency	Percentage
**Gender**					
Male	304	36.7%	Male	268	43.3%
Female	524	63.3%	Female	351	56.7%
**Age**					
18–29	611	73.8%	18–29	415	67%
30–39	150	18.1%	30–39	104	16.8%
40–49	41	5%	40–49	65	10.5%
50–69	26	3.1%	50–69	34	5.5%
70–89	0	0.00%	70–89	1	0.2%
**Governorate/Emirate**					
Ajloun	49	5.9%	Abu Dhabi	303	49%
Amman	128	15.5%	Dubai	221	35.7%
Aqaba	7	0.85%	Sharjah	51	8.2%
Balqa	17	2.05%	Ajman	14	2.3%
Irbid	480	58%	Fujairah	5	0.8%
Jarash	30	3.6%	Ras Al-Khaimah	22	3.6%
Karak	7	0.85%	Umm Al-Quwain	2	0.32%
Maan	2	0.24%	Missing Data	1	0.16%
Madaba	3	0.36%			
Mafraq	32	3.9%			
Tafileh	5	0.6%			
Zarqa	68	8.2%			
**Educational Level**					
Illiterate	-	-	Illiterate	1	0.16%
High school	483	58.3%	High school	113	18.3%
Bachelor’s degree	192	23.2%	Bachelor’s degree	360	58.2%
Master’s degree	84	10.1%	Master’s degree	77	12.4%
PharmD and Ph.D. degrees/Postdoctoral fellowship	69	8.3%	PharmD and Ph.D. degrees/ Postdoctoral fellowship	68	11%
**Have you been diagnosed with COVID-19?**					
No	318	51.4%	No	373	45%
Yes	301	48.6%	Yes	455	55%

**Fig. 1:**
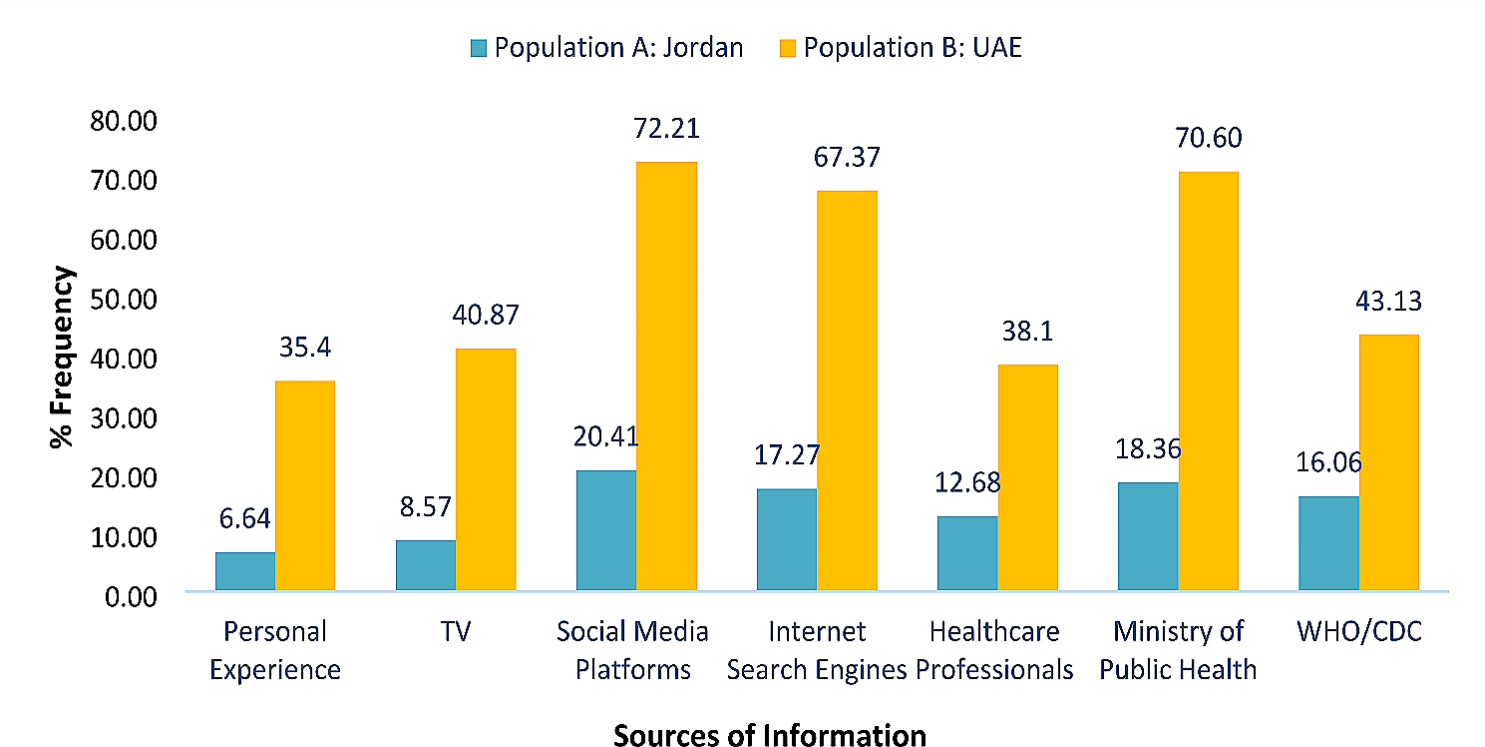
Bar graph representing the respondents’ reliance (% frequency) on different resources of COVID-19 information

Examining the sources of COVID-19 information among the study population (Fig. 1), population B showed a higher motivation (≥ 3 times) to raise their awareness about the preventive measures of COVID-19 than population A. Social media platforms (Jordan: 20.41%; UAE: 72.21%), Ministry of Health (Jordan: 17.27%; UAE: 70.6%), internet search engines (Jordan: 58.8%; UAE: 67.37%), and WHO/CDC webpages (Jordan: 16.06%; UAE: 43.13%) were the most preferred resources of COVID-19 information among the study population (Fig. 1).

### Evaluation of awareness (A) and performance (P)

The evaluation of the community awareness regarding disinfectant use among the two populations is represented in Table 2. Population B recorded somehow higher awareness of disinfectant use than population A regarding A1 (53.2% vs. 43.7%) and A8 (50.2% vs. 9.9%) items. In population A, none of the awareness items were correctly identified by ≥ 50% of the respondents. For example, A1 was more correctly identified by population B than A where 53.2% vs. 43.7% of the respondents consider ethanol as an active substance of skin disinfectant, respectively. Similarly, A8 was more correctly identified by population B than A Table 2).

**Table 2:** Frequencies and percentages of the respondents’ (populations A and B) awareness regarding the use of disinfectants

Awareness	Population A – Jordan: Correct Responses		Population B – UAE: Correct Responses	
	Frequency	Percentage	Frequency	Percentage
**A1** – Which alcohol is used as a disinfectant? (**Ethanol**, Methanol, Both, None, Don’t Know)	317	43.7%	330	53.2%
**A2** – Which one is used for surface disinfection? (Sodium hypochlorite, Perchlorine, Alcohol, **All of the above**, Don’t Know)	173	23.9%	104	16.8%
**A3** – Which alcohol is industrial alcohol that is toxic and deadly? (Ethanol, **Methanol**, Ethanol & Methanol, None, Don’t Know)	223	30.8%	260	41.9%
**A4** – How much chlorine is normally present in bleach? **(5%**, 20%, 70%, 100%, Don’t Know)	47	6.5%	184	29.7%
**A5** – What is the ratio of bleach to water for making surface disinfection? (1 to 2, 1 to 5, 3 to 1, **1 to 50**, Don’t Know)	53	7.3%	75	12.1%
**A6** – For pre-disinfection of fruits and vegetables, how many minutes do they need to be in water and vinegar? (2 to 5, **5 to 15**, 30, 60, Don’t Know)	219	30.2%	168	27.1%
**A7** – What is the recommended ratio of vinegar to water for pre-disinfection of fruits and vegetables? (**1 to 3**, 7 to 10, 15 to 20, 20 to 30, Don’t Know)	199	27.5%	225	36.3%
**A8** – Which one is the most effective concentration of alcohol for skin disinfection? (0.5%, 1%, **70%**, 95%, Don’t Know)	72	9.9%	311	50.2%
**A9** – How long can it take for the disinfectant solution prepared by chlorine to be used for disinfection? (1 h, **1 day**, 1 week, 1 month, Don’t Know)	133	18.3%	86	13.9%
**A10** – At which temperature do you use water to dilute disinfectant solution? (**Room-temperature**, Warm, Hot water, None, Don’t Know)	271	37.4%	107	17.3%

*Options in bold indicate correct answers

The identification of methanol as a toxic and deadly alcohol was less misidentified by population A (76.1%) than population B (83.2%). In addition to skin disinfection, A7 and A8 were inconsistently misidentified among the two populations A and B (Table 2). A2, A9, and A10 are less misidentified among the population A (76.1%, 62.6%, and 81.7%, respectively) than population B (83.2%, 82.7%, and 86.1%, respectively) (Table 2).

**Fig. 2:**
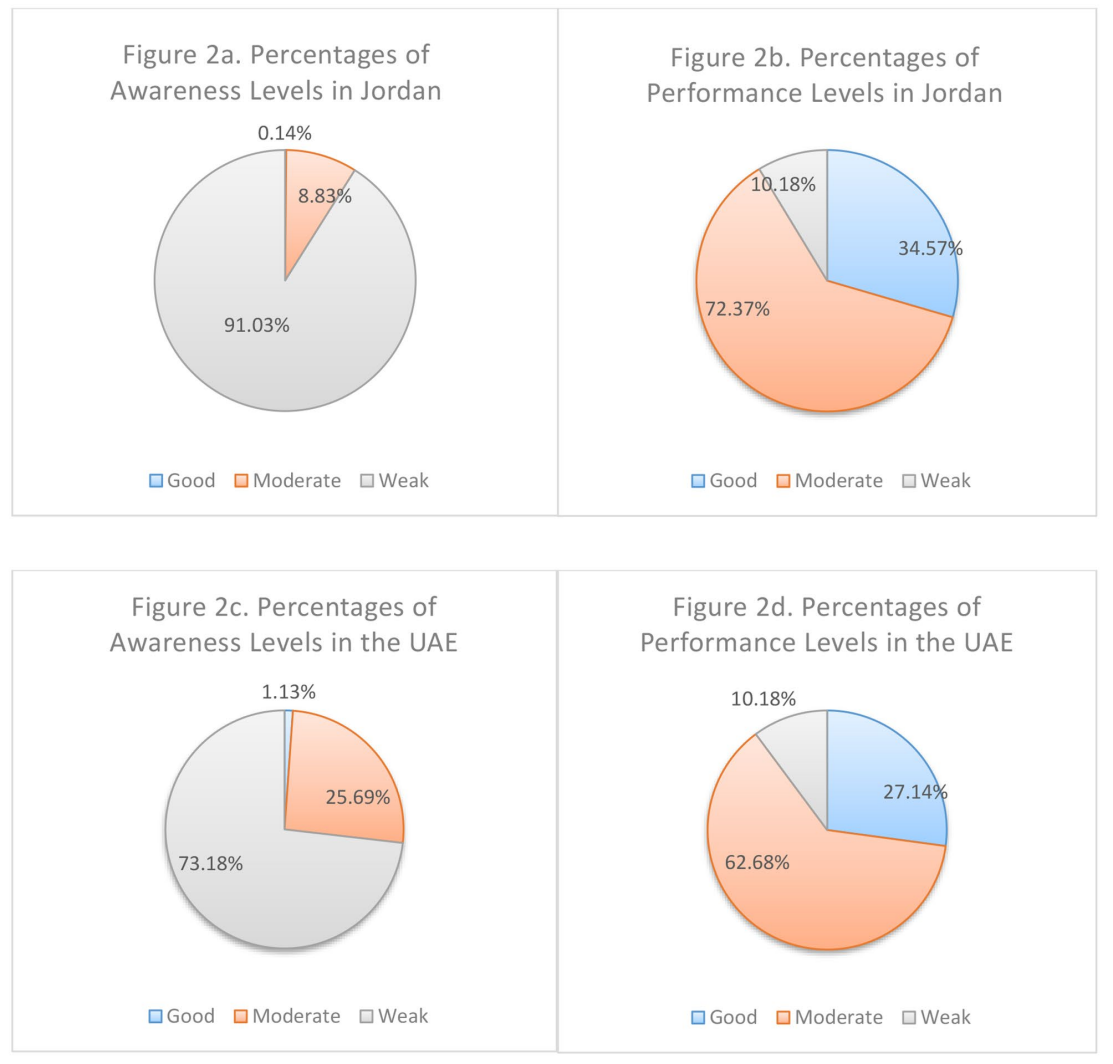
Pie charts representing the % frequency of community levels of awareness in Jordan (**a**), performance in Jordan (**b**) awareness in the UAE (**c**), and performance in the UAE (**d**)

By comparing the two populations (Fig. 2), population B recorded a higher level of moderate-to-good awareness and equal level of moderate-to-good performance. The Spearman correlation test showed that the community awareness and performance were weakly correlated in both of UAE (rho = 0.093, *P* = 0.02 < 0.05) and Jordan (rho = 0.164, *P* < < 0.05).

### Association of awareness and performance with the study population’s characteristics

Comparative analyses between the study population characteristics (Jordan and UAE) and their levels of awareness and performance regarding the safe use of disinfectants are presented in Tables 3 and 4. In Jordan, Kruskal-Wallis tests revealed significant differences in the levels of awareness with respect to age (*P* = 0.012 < 0.05) and the educational level (*P* = 0.013 < 0.05) where the respondents with age group (≥ 40 years old) and postgraduate degrees (Master’s degrees, PharmD degrees, Ph.D. degrees, and postdoctoral fellowships) recorded higher median scores of awareness (3 with IQR of 2–3) (Table 3). The Mann-Whitney test result indicated a significant difference (*P* < < 0.05) in the performance level between males and females (36 with IQR of 13) and Kruskal-Wallis test showed significant differences in performance levels with respect to governorate (*P* = 0.03 < 0.05). The multinomial logistic regression test showed no significant association (*P* > 0.05) between each demographic variable and levels of awareness except for age group (*P* < 0.05) while no significant association (*p* > 0.05) was shown between each demographic variable and levels of performance except for gender in Jordan (*P* < 0.05).

**Table 3:** Statistical association of awareness and performance levels with population A-Jordan characteristics

Variables	Awareness			Performance		
	Median	IQR	*P*-value^*^	Median	IQR	*P*-value^*^
**Gender**						
Male	2.00	2.00	0.82	33.00	14.00	**< 0.001**
Female	2.00	2.00		36.00	13.00	
**Age**						
18–29	2.00	2.00	**0.012**	35.00	13.00	0.098
30–39	2.00	3.00		34.00	13.00	
40–49	3.00	2.00		32.00	17.00	
50–69	3.00	3.00		32.00	16.00	
70–89	-	-		-	-	
**Governorate**						
Ajloun	2.00	2.00	0.13	36.00	13.00	**0.03**
Amman	2.00	2.00		32.00	12.00	
Aqaba	3.00	4.00		29.50	24.00	
Balqa	3.00	2.00		28.00	13.00	
Irbid	2.00	2.00		34.50	14.00	
Jarash	2.00	3.00		39.00	9.00	
Karak	2.00	2.00		36.00	11.00	
Maan	-	-		-	-	
Madaba	3.00	-		39.00	-	
Mafraq	2.00	2.00		38.00	18.00	
Tafileh	3.00	2.00		33.00	11.00	
Zarqa	2.00	2.00		35.00	16.00	
**Educational level**						
Illiterate	-	-	**0.013**	-	-	0.072
High School degree	2.00	2.00		35.00	13.00	
Bachelor’s degree	2.00	2.00		35.00	15.00	
Master’s degree	3.00	2.00		35.00	11.00	
PharmD, Ph.D. degrees and postdoctoral fellowship	3.00	3.00		31.00	19.00	

*Values in bold show significant *P*-values less than 0.05

**Table 4:** Statistical association of awareness and performance levels with population B-UAE characteristics

Variables	Awareness			Performance		
	Median	IQR	*P*-value^*^	Median	IQR	*P*-value^*^
**Gender**						
Male	2.00	3.00	**< 0.010**	33.00	16.00	0.089
Female	3.00	3.00		34.00	15.00	
**Age**						
18–29	3.00	3.00	0.068	33.00	13.00	0.633
30–39	3.00	4.00		33.50	19.00	
40–49	3.00	3.00		33.00	16.00	
50–69	4.00	4.25		37.00	17.00	
70–89	-	-		-	-	
**Emirates**						
Abu Dhabi	3.00	4.00	0.150	34.00	16.00	0.830
Dubai	3.00	3.00		33.00	15.00	
Sharjah	3.00	3.00		33.00	11.00	
Ajman	3.00	2.25		34.50	10.00	
Fujairah	1.00	2.50		34.00	12.00	
Ras Al-Khaimah	4.00	2.00		37.50	15.00	
Umm Al-Quwain	1.50	-		30.00	-	
**Educational level**						
Illiterate	-	-	0.112	-	-	0.732
High School degree	3.00	4.00		33.00	13.00	
Bachelor’s degree	3.00	3.00		34.00	15.00	
Master’s degree	3.00	4.00		34.00	17.00	
PharmD, Ph.D. degrees and postdoctoral fellowship	4.00	3.00		32.00	16.00	

*Values in bold show significant *P*-values less than 0.05

In the UAE (Table 4), Mann-Whitney test indicated that there was a significant difference (*P* < 0.05) in the awareness level between males and females (3 with IQR of 3). All the other variables (age, emirates, and educational level) showed no significant differences (*P* > 0.05) in awareness and performance levels at. The multinomial logistic regression test showed no significant association (*P* > 0.05) between each demographic variable and levels of awareness except for gender (*P* = 0.021 < 0.05) while no significant association (*P* > 0.05) existed between each demographic variable and levels of performance except for educational level (*P* < < 0.05).

Upon comparing if significant differences in the awareness levels occur between the gender in the two populations, the results of the univariate ANOVA (Table 5) indicated an effect of the country on the mean awareness score (P <<< 0.05), a significant difference in the mean awareness score between males and females (*P* = 0.0003 < 0.05) and an interaction of gender and country on the mean awareness score (*P* = 0.002 < 0.05). Regarding the age group, there was an effect of country on the mean awareness scores and a difference in the mean awareness score among the age groups (*P* < < 0.05). Similarly, the same applies for educational level (*P* < 0.05).

**Table 5:** Univariate ANOVA results for the comparison of awareness and performance levels of each of the tested variables between the two populations

Awareness Levels			Performance Levels	
**Gender**				
**Source**	F	*P* ^*^	F	*P* ^*^
Country	37.12	**1.45E-09**	3.121	0.078
Gender	8.86	**0.003**	17.245	**3.49E-05**
Country * Gender	9.56	**0.002**	1.681	0.195
**Age Group**				
**Source**	**F**	**P** ^*^	**F**	**P** ^*^
Country	12.49	**4.23E-04**	0.06	0.81
Age group	5.6	**1.80E-04**	0.91	0.46
Country * Age group	0.03	0.99	1.44	0.23
**Educational Level**				
**Source**	**F**	**P** ^*^	**F**	**P** ^*^
Country	25.5	**5.04E-07**	1.85	0.17
Educatioal level	3.76	**4.78E-03**	1.72	0.14
Country * Educational level	0.65	0.58	0.5	0.69

*Values in bold show significant *P*-values less than 0.05. F statistic value represents the mean square of the variable divided by the mean square of each parameter, while *P* value represents the significane of the F statistic

Upon analyzing whether significant differences existed in performance levels between gender across the two populations, the outcomes from the univariate ANOVA (Table 5) indicated a gender-specific impact on the average performance score only (P <<< 0.05).

## Discussion

To effectively limit the spread of SARS-CoV-2 in Jordan and UAE, this study was designed to shed light on awareness and performance levels among the public communities regarding the safe use of chemical-based disinfectants. To the best of our knowledge, no similar studies have been published in these countries. The finding of this study is to provide the UAE and Jordanian health authorities and their healthcare professionals with new evidence on the frequent use of chemical-based disinfectants and their potential implications on the community health. Additionally, it provides the public health researchers in the Middle East with baseline records to initiate community-based interventions to raise the awareness of the safe use of hygiene-based chemicals and accordingly avoid the health risks of chemical misuse as a part of the United Nations (UN) sustainability goal of wellbeing (Sustainable Development Goal 3, SDG 3).

During the spread of the pandemic, awareness of SARS-CoV-2 in Middle Eastern Arab countries were highly required to guarantee the safe preventive measures especially among the refugees and migrant communities as well as during religious activities. Thereby, local and international organizations have offered a series of awareness campaigns throughout their official webpages to benefit from the public use of the internet services during remote learning and working and to provide the public communities with the reliable information about COVID-19 prevention [22–24]. This study investigated the community reliance on several resources of information to find that the public community in both countries preferred to get their information mostly from the specialized health authorities (Ministry of Health, CDC, and WHO), social media platforms, and internet search engines. Except for the Lebanese Ministry of Public Health (MoPH), the university communities showed a similar preference of the COVID-related resources of information in Lebanon [14]. Thereby, a new concern regarding the unendorsed and misleading information are to be highlighted from the public use of the internet webpages and social media platforms as a guidance to limit the spread of the pandemic [25]. Additionally, electronic resources have either focused on educating people about the preventive measures (wearing a mask, keeping a physical distancing, and regular hygiene with soapy water and/or sanitizers) and symptoms of SARS-CoV-2 infection or tracing contacts and movement via artificial intelligence to enforce quarantine compliance and symptom checking [26] rather than sharing the technical information regarding the safe use of chemical-based disinfectants (e.g. protocols of dilution, storage conditions, and types of chemicals). As a result, governmental authorities and public health organizations are requested to offer a series of technical workshops and awareness campaigns virtually and/or on-site to raise the public awareness of chemical safety for this pandemic era, post-COVID-19, and for the day-to-day use of hygiene products.

The comparison of the awareness levels regarding the safe use of chemical-based disinfectants among the four Middle East countries (Jordan, UAE, Lebanon, and Iran) showed that the community in Iran (48%) recorded higher moderate-to-good level of awareness than that of Lebanon (29.2%), UAE (26.8%), and Jordan (8.97%) [12, 13]. The variation of awareness levels could be derived from the community’s passion to get informed about COVID-19. Except for the Iranian study (where no investigation of the resources of COVID information existed), the higher levels of awareness in Lebanon and UAE may be related to the higher reliance on multiple resources of COVID information than the community in Jordan. Furthermore, the comparison of the community performance recorded almost equal moderate-to-good levels among the communities in Iran, UAE, and Jordan (98%, 98.8%, and 98.8%, respectively) and a slightly higher level of moderate-to-good performance than that of Lebanon (93.8%). In summary, the four Middle Eastern countries recorded higher levels of performance than community awareness. The latter was explained by the structural design of the survey tool which includes technical-based awareness items and basic/scientific-based performance items [14]. Spearman correlation tests showed that community awareness and performance were strongly correlated in the Iranian study (Rho = 0.95, *P* < 0.01) and weakly correlated in Jordan (Rho = 0.164, *P* < 0.05), Lebanon (Rho = 0.14, *P* < 0.05), and UAE (Rho = 0.093, *P* < 0.05). The weak correlation in these three countries may be influenced by the attitude along with the awareness of the study population. Moreover, the attitude of the respondents was not investigated to show its potential impact on community performance.

Similar to the literature studies [11–14], the comparative analyses of awareness levels with respect to sociodemographic variables showed higher awareness scores among the older-age (40 + years old), and the highly educated participants (Master’s degree and above) in Jordan. These findings might be explained by the parenthood role of the older-age participants and the capabilities of the highly educated people to ensure the reliability of resources of information to learn how to safely protect their children from the COVID-19 [13]. The comparative analyses of performance levels with respect to sociodemographic variables showed higher performance scores among females and the residents of some Jordanian governorates (Ajloun, Jarash, Madaba, Karak, and Mafraq). The gender-based finding may be clarified by the fact that females are primarily responsible for taking care of the hygienic conditions of the house and children [27]. In the UAE, gender was the sole notable variable where females recorded a higher awareness score than males as previously confirmed in some literature studies [28, 29]. All the other sociodemographic variables did not record significant differences with respect to the awareness and performance levels to highlight the importance of the comprehensive role of the UAE government especially the execution of detailed awareness campaigns that targeted different groups of the community in response to the pandemic [30].

Among the two populations, the comparative analyses revealed an interaction of the country and gender on the awareness mean scores. This finding highlighted the necessity of designing technical workshops and awareness campaigns that would target both genders of the public community in the UAE and Jordan.

### Study weakness and limitations

In the study design, several limitations are noted. The data collection stage using a self-responding tool may contain a responding bias or social desirability. The online data collection during the pandemic creates another limitation that would weaken the randomness of the study sampling especially for the potential participants who had a lack of familiarity with electronic tools or poor communication connectivity. To overcome the limitations of the online data collection on social media platforms, the e-survey was also distributed among the academic communities via the institutional research offices at the UAE and Jordanian universities. Additionally, the study finding has unequal distribution of gender-based respondents (dominant number of females), educational level-based respondents (dominant number of high school level in Jordan compared to bachelor level in the UAE), and geographical location-based respondents (dominant number of residents in 2 out 12 governorates in Jordan and 2 out of 7 UAE) which may weaken the comparison between the two countries and the extraction of generalized findings for each participating country.

## Conclusions

The study found that the public in the UAE had higher awareness levels (26.8% moderate-to-good) compared to Jordan (8.97%) and equal performance levels (98.8% moderate-to-good in both). Both groups scored higher in performance than awareness, with a weak correlation between the two, indicating the need for awareness campaigns on safe disinfectant use. Age, gender, education, and location influenced awareness and performance differently in each country. In Jordan, those over 40 and with postgraduate degrees showed better awareness, while in the UAE, female participants scored higher. For performance, Jordanian females and residents of certain areas scored positively, unlike in the UAE. The study recommends community-based interventions, risk assessments, toxicological studies, awareness campaigns, and technical workshops to address disinfectant misuse in the Middle East.

### Electronic supplementary material

Below is the link to the electronic supplementary material.

Supplementary Material 1

## Data Availability

The datasets used and/or analyzed during the current study are available from the corresponding authors on reasonable request.

## Abbreviations

COVID-19: Coronavirus Disease 2019
SARS-CoV-2: Severe Acute Respiratory Syndrome Coronavirus-2
WHO: World Health Organization
CDC: Centers for Disease Control and Prevention
UAE: United Arab Emirates
MoPH: Ministry of Public Health
UN: United Nations
SDG: Sustainable Development Goal
